# Role Assessment of Water-Soluble Pharmaceutical Form of Phosphatidylcholine on the Catalytic Activity of Cytochrome P450 2C9 and 2D6

**DOI:** 10.3390/ijms26010004

**Published:** 2024-12-24

**Authors:** Polina I. Koroleva, Tatiana V. Bulko, Alexey V. Kuzikov, Andrei A. Gilep, Yulia A. Romashova, Elena G. Tichonova, Lyubov V. Kostrukova, Alexander I. Archakov, Victoria V. Shumyantseva

**Affiliations:** 1 Institute of Biomedical Chemistry, Pogodinskaya Street, 10, Build 8, 119121 Moscow, Russiaalexeykuzikov@gmail.com (A.V.K.); kostryukova87@gmail.com (L.V.K.);; 2 Faculty of Biomedicine, Pirogov Russian National Research Medical University, Ostrovitianov Street, 1, 117997 Moscow, Russia

**Keywords:** cytochrome P450 2C9, cytochrome P450 2D6, electrochemical analysis, naproxen, bisoprolol, phosphatidylcholine, macromolecular crowding

## Abstract

This study aimed to investigate whether the water-soluble pharmaceutical form of phosphatidylcholine nanoparticles (wPC) stimulated the catalytic activity of CYP enzymes 2C9 and 2D6. We have shown that electroenzymatic CYP2C9 catalysis to nonsteroidal anti-inflammatory drug naproxen as a substrate was enhanced from 100% to 155% in the presence of wPC in media. Electroenzymatic CYP2D6 activity in the presence of the adrenoceptor-blocking agent bisoprolol as a substrate was elevated significantly from 100% to 144% when wPC was added to potassium phosphate buffer solution. These results indicate the ability of wPC in the form of the phospholipid ultra-small nanoparticles to work as a membrane additive and crowding agent to accelerate the electroenzymatic reactions of cytochrome P450.

## 1. Introduction

Cardiovascular disease (CVD) and atherosclerotic cardiovascular disease (ASCVD) are major causes of morbidity and global mortality. Recent works have confirmed that the ratio of high-density lipoprotein cholesterol (HDL-C) is in strong interdependence with the probability of CVD occurrence. Clinical trials have shown the correlation between dyslipidemia and CVD/ASCVD [1,2]. Therapeutic drugs that lower low-density lipoprotein cholesterol possess advantages as cardioprotectors. The principal features of HDL particles are their anti-inflammatory effects, the prevention of thrombus formation and the backward transport of cholesterol molecules, which makes them associated with CVD and an extended lifespan [3].

The water-soluble pharmaceutical form of a phospholipid used here was ultra-small phospholipid nanoparticles from soybean phosphatidylcholine (wPC), with an average size of 30 nm [3]. In an in vitro study, it was shown that there was a dose-dependent eight-fold increase of wPC in HDL, and a two-fold increase in the cholesterol efflux capacity, as compared with native apo-B-depleted plasma, when incubated with phospholipid nanoparticles (PL) [3,4]. In an in vivo study, it was found that the intravenous administration of wPC nanoparticles in the cholesterol-fed rabbits restored lipid droplets, protected the vessel wall from developing intimal lipid lesions, and showed a similar decrease in the extent of atherosclerotic lesions, as compared with atorvastatin and fenofibrate [5,6]. Combined hyperlipidemia is associated with an increased risk of cardiovascular events. Clinical studies obviously demonstrated the lowering of non-high-density lipoproteins, cholesterol, and triglyceride levels in patients with combined hyperlipidemia and moderate cardiovascular risk after 12 weeks of treatment of wPC with 1 g daily [3]. However, long-term drug use as a stress factors may influence not only the cholesterol and triglycerides levels, but reduce or enhance the catalytic activity of vital objects such as enzymes. Cytochrome P450 (CYP) is the main membrane enzyme responsible for the metabolism of exogenous compounds as xenobiotics, drugs, toxins, and the biosynthesis of endogenous compounds—for example, steroids, fatty acids, vitamins. Human CYP enzymes are responsible for 75–80% of all phase-I drug metabolism [7,8,9,10]. The unique properties of cytochrome P450 as a biocatalyst include its high diversity of endobiotic and xenobiotic metabolic pathways. In biotechnological applications, the regioselectivity, stereoselectivity and broad substrate landscape of the P450 enzymes have stimulated extensive efforts to engineer P450 systems to overcome the intrinsic limitations of the native enzymes [7,8,9,10,11,12]. The activity of membrane CYP enzymes strongly depends on the membrane composition [13,14,15]. The membrane environment affects the substrate interaction with the enzyme active site [15]. The endoplasmic reticulum contains over 50% phosphatidylcholine (PC) and 20% phosphatidylethanolamine [16,17,18]. Modulation by cell membrane composition and phosphatidylcholine amounts in biological systems was conformed in [15]. Lipid composition and, in addition, macromolecular crowding, are key external effectors of protein activity [18]. The internal space of the cell is characterized by a high degree of crowding, provided by proteins, lipids, nucleic acids, polysaccharides, and small molecules. For example, *Escherichia coli* contains about 350 mg/mL of various proteins and RNA, whereas mammalian cells consist of 20–30% macromolecules [17]. Buffer solutions do not reflect the complex composition of cell media [19].

To mimic the crowded membrane environment, we used the water-soluble pharmaceutical form of phosphatidylcholine from soybean (wPC). We tested the influence of wPC on the catalytic activity of CYP2C9 and 2D6 enzymes. wPC was investigated as a crowding additive and in model membrane composition during the construction of an electrochemical CYP-biosensor and CYP-nanobioreactor with the P450 2C9 and 2D6 forms of these enzymes. We have shown that PC in electrolyte solution significantly enhanced the catalytic activity of both CYPs, regarding naproxen as a substrate of CYP2C9 [20] and bisoprolol as a substrate of CYP2D6 [21].

For the analysis of the CYP2C9-dependent O-demethylation of naproxen, the registering of formaldehyde formation was carried out [22,23]. For the analysis of bisoprolol O-hydroxylation, we used a previously elaborated approach based on the difference in the amplitude of substrate electrochemical oxidation before electrolysis at an applied potential and after electrolysis during 30 min using a screen-printed electrode modified with carbon nanotubes (SPE/CNT) [24].

Cytochrome P450 2C9 (CYP2C9) ranks second in expression among the liver cytochrome P450 enzymes, and accounts for approximately 20% of the complete cytochrome P450 isozyme content in the liver. CYP2C9 is the second of the major CYP isozymes, which metabolizes about 15–20% of drugs, especially drugs with a low therapeutic index that undergo Phase I metabolism by CYP2C9. A number of these drugs are non-steroidal anti-inflammatory (diclofenac, ibuprofen, naproxen, celecoxib, piroxicam), antiagregant (acenocoumarol, S-warfarin), antihypertensive (candesartan, losartan), hypolipidemic (fluvastatin), antidiabetic (glyburide, tolbutamide), dissociative anesthetic (ketamine), opioid (methadone), anticonvulsant (phenytoin, phenobarbital), and antitumor (tamoxifen) drugs. In addition, CYP2C9 is responsible for the metabolism of endogenous compounds such as steroid hormones, vitamin A, and fatty acids [8,9,10]. Enzymes from the CYP2D subfamily actively interact with xenobiotics containing a nitrogen atom protonated at a physiological pH [9,10].

Electrochemical systems allowing the implementation of transport of electrons between the working electrode surface and the enzyme active center are useful for understanding the electrocatalytic mechanism, as well as designing an efficient assay system for the investigation of drug–drug interactions, biocatalysis, the screening of substrates and inhibitors, and the construction of bioreactors. Electrochemically driven catalysis with electrodes as the electron donor allow for the creation of effective cytochrome P450 catalytic systems [18,22,23,24,25]. CYP-based sensors are helpful instruments for the assessment of inducers or activators of CYP-dependent catalytic activities [15,25,26].

To our knowledge, no studies investigating the influence of ultra-small phosphatidylcholine nanoparticles (wPC) on the catalytic activity of cytochrome P450 have been reported yet. Therefore, in this paper, we investigated the metabolic transformations of naproxen as a substrate of CYP2C9 and bisoprolol as a substrate CYP2D6. The positive effects of ultra-small phosphatidylcholine nanoparticles as a crowding reagent and in model membrane composition in electrolyte media were registered.

## 2. Results and Discussion

### 2.1. Characterization of the CYP2C9 Electrochemical System

The cytochrome P450 family are oxidoreductases, so they need electron donors to carry out the catalytic process; in cells or reconstructive systems, redox partner proteins perform this function. Therefore, electrochemical systems are used to model the catalytic process in vitro [22,23,24,25], which has proven their effectiveness in searching for substrates and inhibitors, as well as in assessing drug–drug interactions [26,27,28,29].

Our group, in detail, for the aerobic and anaerobic conditions, earlier described an investigation of the electrochemical properties of CYP2C9 immobilized on screen-printed electrode-modified DDAB (SPE/DDAB/CYP2C9) [26,30]. In the argon-saturated buffer SPE/DDAB/CYP2C9, two peaks (E_a_ = −0.252 ± 0.006 V, E_c_ = −0.383 ± 0.01 V) were observed, which were attributed to the reversible redox reaction of heme iron according to the scheme Fe (III) + e ↔ Fe(II), corresponding to a non-catalytic direct redox reaction [31]. The midpoint potentials E^0^′ were determined as half of the sum of the means of the potentials of the anodic and cathodic peak. CYP2C9 demonstrated a midpoint potential value of E^0′^ = −0.318 ± 0.03 V [27,28]. A linear dependence of the maximum amplitudes of the cathodic and anodic currents on the scanning speed [24,30,31] characterized the adsorbed redox pairs. When oxygen is present in the system, the reduced Fe(II) ion irreversibly binds the oxygen molecule, and the cathode current corresponds to the reaction CYP-Fe (II) + O_2_ → CYP-Fe (II) O_2_, and the current value is in positive correlation with the scan rate [27].

During the catalytic cycle, cytochrome P450, as a monooxigenase, binds an oxygen molecule, and this event occurs only after the formation of the enzyme substrate complex [8,9,10,11,12]. In our model electrochemical CYP2C9 system, we used naproxen as substrate of the CYP2C9 enzyme. Naproxen (6-methoxy-α-methyl-2-naphthaleneacetic acid) is a nonsteroidal anti-inflammatory drug. Naproxen inhibits cyclooxygenases 1 and 2 and is commonly used to treat arthritis, ankylosing spondylitis, tendinitis, bursitis, and gout. It was shown that naproxen and its derivatives are effective against various tumors [32,33,34].

To assess the catalytic efficiency of the enzyme on the electrode, the ratio of the maximum amplitude of the catalytic current of the CYP-bioelectrode in the presence of the substrate to the reduction current (I_cat_/I_O2_), called the catalytic or coupling index, was used. In addition, this ratio shows the distribution of the electron flow and allows us to evaluate the coupling efficiency. For SPE/DDAB/CYP2C9 versus 100 μM naproxen, the I_cat_/I_O2_ was 2.04. The electrochemical system SPE/DDAB/CYP2C9 bioelectrode was characterized with a potential of catalysis Ecat = −0.432 ± 0.024 V, a potential of heme reduction Ered = −0.495 ± 0.015 V, and a potential at the start of the catalysis Eonset = −0.266 ± 0.036 V (Table 1). The analytical sensitivity of the bio-electrode SPE/DDAB/CYP2C9 in relation to the naproxen was 0.0034 A/M (S/N = 3).

Phospholipid nanoparticles can be used for two purposes [1,2,3,4,5]:

1. As pharmacologically active drugs that restore the structural integrity and functional activity of biological membranes and high-density lipoproteins;
2. As a transport system for hydrophobic drugs, converting them from water-insoluble to water-soluble compounds and ensuring their delivery in increased quantities to target organs by improving pharmacokinetics.

The cytochrome P450 (CYP) family are the main membrane enzymes responsible for the detoxification of xenobiotics [8,9,10,11,12]. The influence of water-soluble ultra-small phosphatidylcholine nanoparticles (wPC) on the electroenzymatic activity of CYP2C9 was investigated.

For the analysis of the influence of wPC on CYP2C9 catalysis, using naproxen as a substrate, cyclic voltammetry was the method of choice. To model the natural membrane and cell surroundings, we used wPC in a solution of electrolyte PBS buffer. The second method was to apply 4 μL of wPC layer-by-layer onto a modified DDAB electrode.

**Figure 1:**
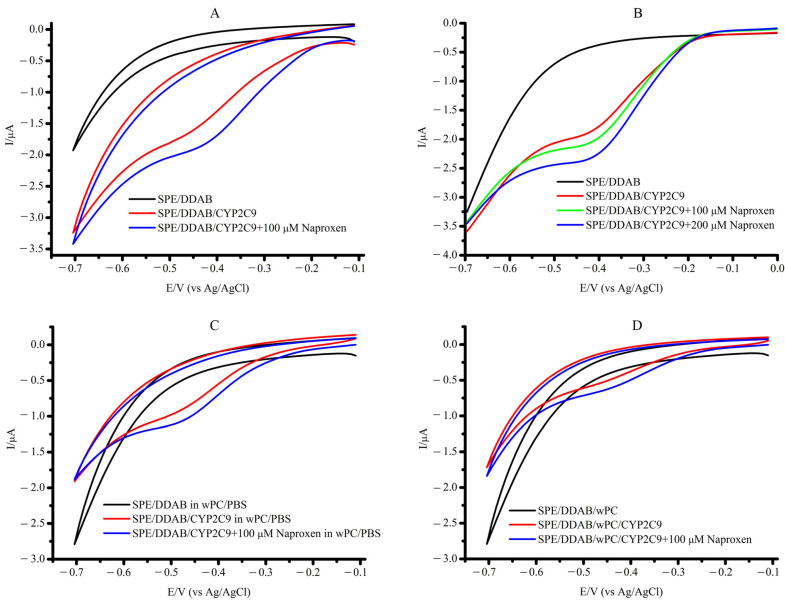
(**A**) Cyclic voltammograms of SPE/DDAB, SPE/DDAB/CYP2C9, and SPE/DDAB/CYP2C9 with 100 μM of naproxen in PBS. (**B**) Square wave voltammograms of SPE/DDAB, SPE/DDAB/CYP2C9, and SPE/DDAB/CYP2C9 with 100 μM of naproxen and 200 μM of naproxen in PBS. (**C**) Cyclic voltammograms of SPE/DDAB, SPE/DDAB/CYP2C9, and SPE/DDAB/CYP2C9 with 100 μM of naproxen in PBS with wPC. (**D**) Cyclic voltammograms of SPE/DDAB/wPC, SPE/DDAB/wPC/CYP2C9, and SPE/DDAB/wPC/CYP2C9 with 100 μM of naproxen in PBS. Cyclic voltammograms were carried out at a scan rate of 0.1 V/s.

The electrochemical and electrocatalytic parameters of SPE/DDAB/CYP2C9 were studied and are represented in Table 1 and Figure 1. CYP2C9 effectively interacted with the substrate naproxen, with a clear registration of a catalytic current by means of cyclic voltammetry and square wave voltammetry (Figure 1A,B). In wPC media, we registered the anodic shift in the CYP2C9 reduction potential Ered from −0.495 ± 0.015 V to −0.465 ± 0.022 V (Figure 1C). When wPC was applied onto the DDAB layer on the surface of the working electrode, the Ered was registered as −0.437 ± 0.017 V (Figure 1D). However, the coupling index I_cat_/I_red_, which permits the evaluation of the efficiency of interactions between CYP2C9 and naproxen, varied slightly across all the systems studied.

**Scheme 1:**
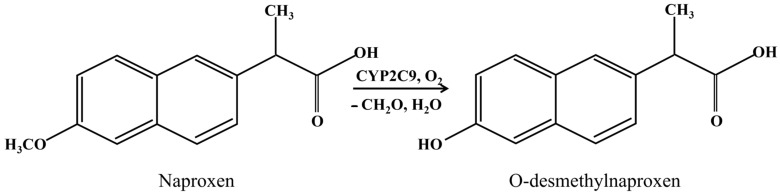
Metabolic transformation of naproxen catalyzed by CYP2C9 [19].

The assessment of the catalytic activity of CYP2C9 towards naproxen in an electrochemical reaction occurring at an applied potential of E = −0.6 V for 60 min was determined by assessing the concentration of the reaction by-product, formaldehyde (Scheme 1), using the Nash reagent based on a Hantzsch reaction at a wavelength of 412 nm [22,23]. The catalytic activity of SPE/DDAB/CYP2C9 in respect to naproxen in wPC-containing electrolyte buffer was evaluated to range from Vmax = 6.44 ± 1.17 × 10^−11^ M/min to Vmax = 9.98 ± 0.69 × 10^−11^ M/min (by 1.5 times), respectively (Table 1). When wPC was inserted into the DDAB layer, this parameter increased and corresponded to 1.04 ± 0.19 × 10^−10^ M/min. This phenomenon may be explained in terms of the close modeling of microsomes’ membrane composition [15,16].

The presence of phospholipids in the electrolyte medium promotes stabilization and an increase in the catalytic activity of hemoproteins, despite a decrease in the amount of electroactive protein on the electrode (Figure 1A,C,D).

### 2.2. Characterization of the CYP2D6 Electrochemical System

Cytochrome P450 2D6 (CYP2D6) is also one of the most important CYP isoenzymes for drug metabolism and the biotransformation of endogenous compounds. Under the action of CYP2D6, many of the most important drugs are converted, including antipsychotics, antidepressants, opioids and antiarrhythmic agents; in total, about 25% of drugs are metabolized by CYP2D6 [35,36].

**Figure 2:**
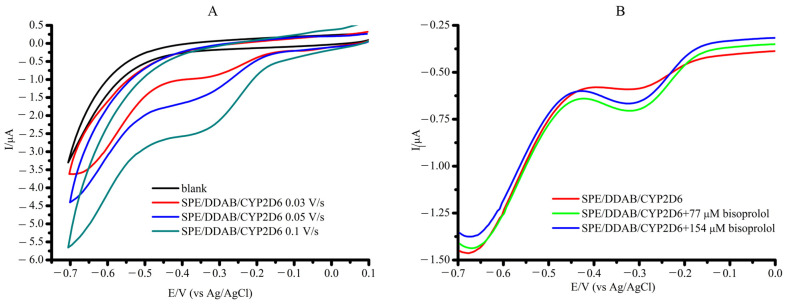
(**A**) Cyclic voltammograms of SPE/DDAB/CYP2D6 in oxygenated electrolyte buffer at a scan rate of 0.03 V/s, 0.05 V/s, and 0.1 V/s. (**B**) SWV of SPE/DDAB/CYP2D6 in oxygenated electrolyte buffer, in the presence of 77 μM bisoprolol and in the presence of 154 μM bisoprolol.

The characterization of the electrochemical system based on CYP2D6 supported on a membrane-like synthetic matrix formed by DDAB on a screen-printed graphite electrode (SPE/DDAB/CYP2D6) was obtained under aerobic conditions. Figure 2A shows the cyclic voltammograms (CV) of CYP2D6 at scan rates of 30, 50, and 100 mV/s. The process of surface electron transfer (“voltammetry of protein films”) was observed, since the maximum current amplitudes were linearly dependent on the scanning speed [29,30]. The electroanalytical characteristics of CYP2D6 are presented in Table 1.

**Scheme 2:**
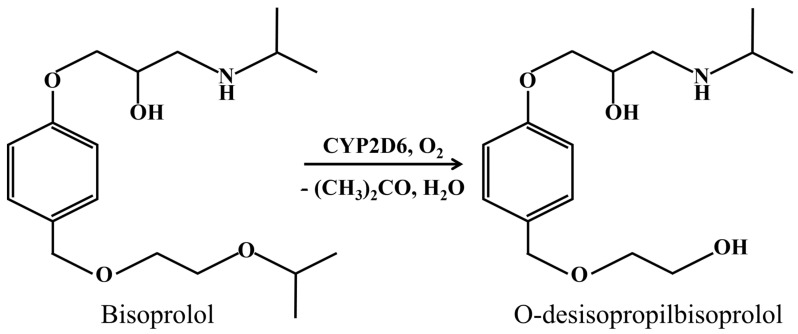
Chemical structure of bisoprolol and its metabolite [1-[4-[[2-(1-methylethoxy]methyl]phenoxy]-3-[(1-methylethyl)amino]-2-propanol (O-desisopropilbisoprolol).

Bisoprolol hemifumarate, [1-[4-[[2-(1-methylethoxy]methyl]phenoxy]-3-[(1-methylethyl)amino]-2-propanol hemifumarate, is a highly selective adrenoceptor-blocking agent and is widely used in therapeutic practice [21]. Bisoprolol hemifumarate undergoes dealkylation under the action of CYP2D6 as shown in the Scheme 2.

The catalytic current corresponding to the interaction between CYP2D6 and bisoprolol was registered with square-wave voltammetry (SWV), reflecting the catalytic activity and sensitivity of CYP2D6 to this drug molecule. As can be seen from Figure 2B, this parameter was concentration-dependent and increased when 77 μM and 154 μM of substrate was used.

**Figure 3:**
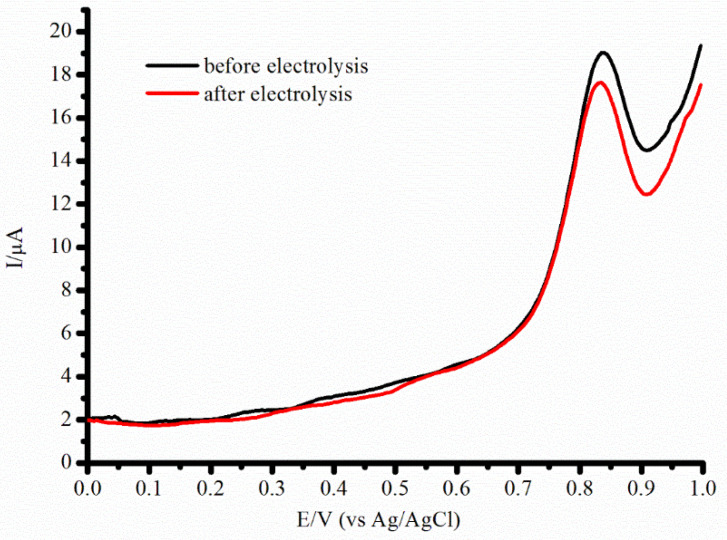
Square-wave voltammetry of SPE/CNT/bisoprolol before electrolysis and after electrolysis at the controlled potential E = −0.5 V, 30 min using SPE/DDAB/wPC/CYP2D6.

**Figure 4:**
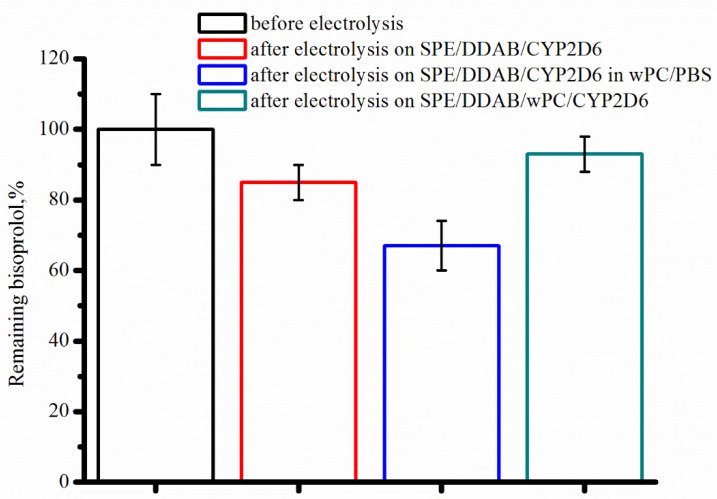
Diagrams corresponding to relative oxidation current of bisoprolol before and after electrolysis at the controlled potential E = −0.5 V, 30 min in electrolyte buffer, in electrolyte buffer with wPC (wPC/PBS), and wPC in a layer of DDAB (DDAB/wPC).

The SWV technique using SPE, modified with carbon nanotubes (SPE/CNT), was applied for bisoprolol determination. To measure the residual concentration of bisoprolol in the probe after cytochrome CYP2D6-dependent electrocatalysis, the peculiarity of bisoprolol electrooxidation at a positive potential was used (Figure 3). Measuring the current of electrochemical oxidation of bisoprolol in solution before and after electrolysis at a controlled potential (E = −0.5 V) for 30 min, allowed us to calculate the catalytic activity of systems in PBS as an electrolyte buffer simulating physiological environments, and in the presence of wPC in a PBS medium (wPC/PBS). The comparative activities of CYP2D6 to bisoprolol in electrolyte buffer, in electrolyte buffer with wPC, and wPC in a layer of DDAB are shown in Figure 4.

The water-soluble pharmaceutical form of phosphatidylcholine in electrolyte PBS significantly enhanced the metabolic activity of CYP2D6 towards bisaprolol from 15% to 23% (Figure 4).

An analysis of the electrocatalytic conversion of bisoprolol in the presence of wPC revealed the influence of this additive on drug metabolism catalyzed by CYP2D6 (Table 1, Figure 4). The positive effects of ultra-small phosphatidylcholine nanoparticles as a crowding reagent and in model membrane composition in electrolyte media on the electroenzymatic activity of CYP2D9 was registered. Differences found in the wPC location in solution or in a lipid-like DDAB membrane underline the specificity of this water-soluble pharmaceutical form of phospholipid (wPC) from soybean phosphatidylcholine as a CYP-stimulating agent.

## 3. Materials and Methods

Human recombinant CYP (175 µM CYP2C9 stock solution in 550 mM potassium phosphate buffer (pH 7.2), containing 0.2% CHAPS, 1 mM dithiothreitol and 20% glycerol (*v*/*v*)) was purified according to the protocol in [37] at the Institute of Bioorganic Chemistry (Minsk, Belarus). CYP2D6 (50 µM stock solution, cat No. C9195) was obtained from Sigma–Aldrich (2D6 isozyme, without P450 reductase, recombinant, expressed in *E. coli*, >85% (SDS-PAGE), ≥50 units/mg protein). The concentration of CYP2C9 or CYP2D6 was determined by complex formation of the reduced form with carbon monoxide using the absorption coefficient ɛ_450_ = 91 mM^−1^cm^−1^ [38]. Chloroform, didodecyldimethylammonium bromide (DDAB), disodium phosphate, and naproxen were obtained from Sigma–Aldrich (USA). Potassium hydroxide, potassium dihydrogen phosphate, and sodium chloride were purchased from Spectrochem (Moscow, Russia). Water dispersion of 0.2% single wall carbon nanotubes (SWCNT, diameter 1.6 ± 0.4 nm, length ≥ 5 μm, surface area 1000 m^2^/g) TUBALL™ BATT H_2_O, stabilized by carboxymethylcellulose, was obtained from OCSIAL Ltd (Novosibirsk, Russia).

In experiments, we used the water-soluble pharmaceutical form of PL nanoparticles as ultra-small phospholipid micelles from soybean phosphatidylcholine, with an average size of 30 nm (wPC), which contained 0.5 g of phosphatidylcholine and 2 g of D-maltose [3,4,5] as a stock solution in 100 mL of 0.1 M potassium phosphate pH 7.4 containing 0.05 M NaCl (PBS). Bisoprolol was purchased from the Vertex pharmaceutical company as a 25 mg of drug/tablet.

All electrochemical experiments were carried out at room temperature (25 °C) and in PBS using a potentiostat/galvanostat PGSTAT 302N Autolab (Metrohm Autolab, Utrecht, The Netherlands), controlled by NOVA software (version 2.0) and an AutoLab 12 potentiostat/galvanostat (Metrohm Autolab, Utrecht, The Netherlands) equipped with GPES software (version 4.9.7).

Screen-printed electrodes (SPE), which consist of the graphite working electrode (surface area 0.0314 cm^2^), an auxiliary electrode, and a silver/silver chloride pseudo reference electrode, were used and obtained from ColorElectronics, Moscow, Russia.

The analysis of cytochrome P450 2C9 and 2D6 electrochemical activity was carried out with electrode modification with the synthetic lipid-like compound DDAB; 0.1 M DDAB in chloroform (1 μL) was applied at the surface of the working electrode, and then electrodes were left for 10 min until completely dried. Immobilization of the enzyme was carried out as described earlier in [25,26], but in this case, we used 1 µL of 175 µM CYP2C9 solution or 2 µL of 50 µM CYP2D6.

All potentials were referenced against the silver/silver chloride reference electrode (Ag/AgCl). Enzymatic electrocatalytic reactions in the presence of the substrate naproxen under aerobic conditions were performed at room temperature in air-saturated buffer; a potential of E = −0.6 V was applied for 60 min.

Cyclic voltammograms (CV) were recorded using a 1 mL electrochemical cell by potential sweeping from an initial potential of −0.1 V to an end-point potential of −0.6 V at different scan rates in a range of 0.01–0.1 V s^−1^. Square-wave voltammograms (SWV) in the 0–1.2 V potential range were registered at a 25 Hz frequency, 40 mV amplitude, and 5 mV potential step. The obtained voltammograms were smoothed and baseline-corrected by the software of the potentiostats. All experiments were performed in triplicate. The data are presented as average values ± standard deviations (±SD). The analysis of the reaction rate of naproxen O-demethylation was carried out by registering formaldehyde formation as described previously [27,28].

The analysis of the reaction rate of bisoprolol 4- hydroxylation with 4-hydroxypropranolol formation was carried out by registering the electrochemical oxidation signal of the bisoprolol before (0 min of electrolysis) and after electrolysis (30 min at the applied potential of E = −0.5 V) with the SWV technique and SPE, modified with carbon nanotubes (SPE/CNT). The surface of the SPE working electrode was coated as described previously [20]. Experiments were conducted under aerobic conditions at room temperature in a horizontal mode.

## 4. Conclusions

Different factors can contribute to the activity of the cytochrome P450 family [26,39,40]. We can assume that the intake of wPC influences the catalytic activity of cytochrome P450 enzymes. Based on our experimental data, we can predict increased metabolic profiles of drugs that act as substrates of CYPs in the presence of wPC. In our experiments, water-soluble ultra-small phosphatidylcholine nanoparticles influenced the CYPs through different mechanisms. wPC exerts its activity as a modulating and/or stimulating additive with respect to CYPs’ catalytic activity, due to its ability to act as a membrane stabilizing and crowding agent.

The regulation and modulation of cytochrome CYP2C9 and 2D6 activates through the action of wPC upon their administration in a combination with clinical drugs metabolized by P450, which will probably become an essential requirement in clinical routine practice. Long-term drug intake can lead to alterations in pharmacodynamics/pharmacokinetic efficiency, which demands special attention from physicians since the prescribed medical product can bring about changes in efficiency/safety profiles. Cytochrome P450 bioelectrodes are indispensable tools, and adequate non-invasive experimental models for new drug development and safe therapies [10,18,22,23,24,25,26,31,32,33]. Electrochemically driven CYP catalysis and CYP-biosensors are effective model systems for pharmacological research and medical applications.

The importance of non-invasive cytochrome P450-based testing system elaboration is dealing with the functional significance of these drug-metabolizing enzymes. Artificial CYP systems may be constructed based on different approaches, such as reconstitution systems, which contain enzyme and protein redox partners, photo-activated systems, or electrochemical systems [13]. The main limitation of artificial CYP-based models is the time-dependent inactivation of cytochrome P450-based systems [8]. This property of CYPs requires disposable electrodes (in the case of an electrochemical system). Effective, non-invasive cytochrome P450-based systems are necessary for the investigation of drug–drug interactions, and in the search for and investigation of new chemical compounds as potential substrates of cytochrome P450 enzymes. Based on the obtained results, we can conclude that electrochemical cytochrome P450-based biosensors are a promising method for the investigation of the catalytic activity of enzymes towards substrates, inhibitors, or modulators/activators.

## Figures and Tables

**Table 1:** Electrochemical and electrocatalytic parameters of SPE/DDAB/CYP2C9 and SPE/DDAB/CYP2D6 in phosphate buffer and phosphate buffer with wPC and SPE/DDAB/wPC/CYP2C9, under aerobic conditions and in the presence of 100 µM naproxen.

System	E_red_, V	E_cat_, V	E_onset_, V	I_cat_/I_red_	Vmax, M/min	Relative Activity, %
SPE/DDAB/CYP2C9	−0.495 ± 0.015	In the presence of 100 µM naproxen				
		−0.432 ± 0.024	−0.266 ± 0.036	2.04	6.44 ± 1.17 × 10^−11^	100 ± 18
SPE/DDAB/CYP2C9 in wPC/PBS	−0.465 ± 0.022	−0.466 ± 0.021	−0.288 ± 0.032	1.67	9.98 ± 0.69 × 10^−11^	155 ± 9
SPE/DDAB/wPC/ CYP2C9	−0.437 ± 0.017	−0.422 ± 0.008	−0.254 ± 0.024	1.91	1.04 ± 0.19 × 10^−10^	161 ± 18
SPE/DDAB/ CYP2D6	−0.373 ± 0.010	In the presence of 77 µM Bisoprolol				
		−0.383 ± 0.015	−0.389 ± 0.015	1.31	0.40 ± 0.10 × 10^−6^	100 ± 15
SPE/DDAB/CYP2D6 in wPC/PBS	−0.323 ± 0.009	−0.353 ± 0.010	−0.383 ± 0.010	2.61	0.57 ± 0.10 × 10^−6^	144 ± 8
SPE/DDAB/wPC/ CYP2D6	−0.313 ± 0.008	−0.323 ± 0.009	−0.365 ± 0.009	1.76	0.17 ± 0.09×10^−6^	47 ± 12

## Data Availability

The data are available upon reasonable request from the corresponding author.
